# Pristimerin‐induced uveal melanoma cell death via inhibiting PI3K/Akt/FoxO3a signalling pathway

**DOI:** 10.1111/jcmm.15249

**Published:** 2020-04-29

**Authors:** Fengxia Yan, Rifang Liao, Marta Silva, Shuai Li, Yizhou Jiang, Tangming Peng, Philip Lazarovici, Wenhua Zheng

**Affiliations:** ^1^ Faculty of Health Sciences University of Macau Macau China; ^2^ School of Medical Science Jinan University Guangzhou China; ^3^ Department of pharmacy Sun Yat‐sen Memorial Hospital Sun Yat‐sen University Guangzhou China; ^4^ Faculty of Medicine School of Pharmacy Institute for Drug Research The Hebrew University of Jerusalem Jerusalem Israel

**Keywords:** cell death, FoxO3a, nuclear accumulation, Pristimerin, uveal melanoma

## Abstract

Uveal melanoma (UM) is a highly invasive intraocular malignancy with high mortality. Presently, there is no FDA‐approved standard for the treatment of metastatic UM. Pristimerin is a natural quinine methide triterpenoid compound with anti‐angiogenic, anti‐cancer and anti‐inflammatory activities. However, Pristimerin potential cytotoxic effect on UM was poorly investigated. In the present study, we found the migration and invasion of UM‐1 cells were inhibited by Pristimerin which also caused a rapid increase of ROS, decreased mitochondrial membrane potential, induced the accumulation of cells in G0/G1 phase, ending with apoptotic cell death. Pristimerin inhibited Akt and FoxO3a phosphorylation and induced nuclear accumulation of FoxO3a in UM‐1 cells, increased the expression of pro‐apoptotic proteins Bim、p27^Kip1^, cleaved caspase‐3, PARP and Bax, and decreased the expression of Cyclin D1 and Bcl‐2. LY294002 or Akt‐siRNA inhibited the PI3K/Akt/FoxO3a pathway and promoted the Pristimerin‐induced apoptosis, while Pristimerin effects were partially abolished in FoxO3a knockdown UM‐1 cell cultures. Taken together, present results showed that Pristimerin induced apoptotic cell death through inhibition of PI3K/Akt/FoxO3a pathway in UM‐1 cells. These findings indicate that Pristimerin may be considered as a potential chemotherapeutic agent for patients with UM.

## 1 INTRODUCTION

Uveal melanoma (UM) is a common primary intraocular malignancy with an annual incidence of about 7 per million.^1^ However, the mechanism underlying UM pathology is unclear. At present, there is no definite treatment for UM. Therefore, it is an important clinical need to study its underlying pathological mechanisms and formulate corresponding chemotherapeutic strategies.

**FIGURE 1:**
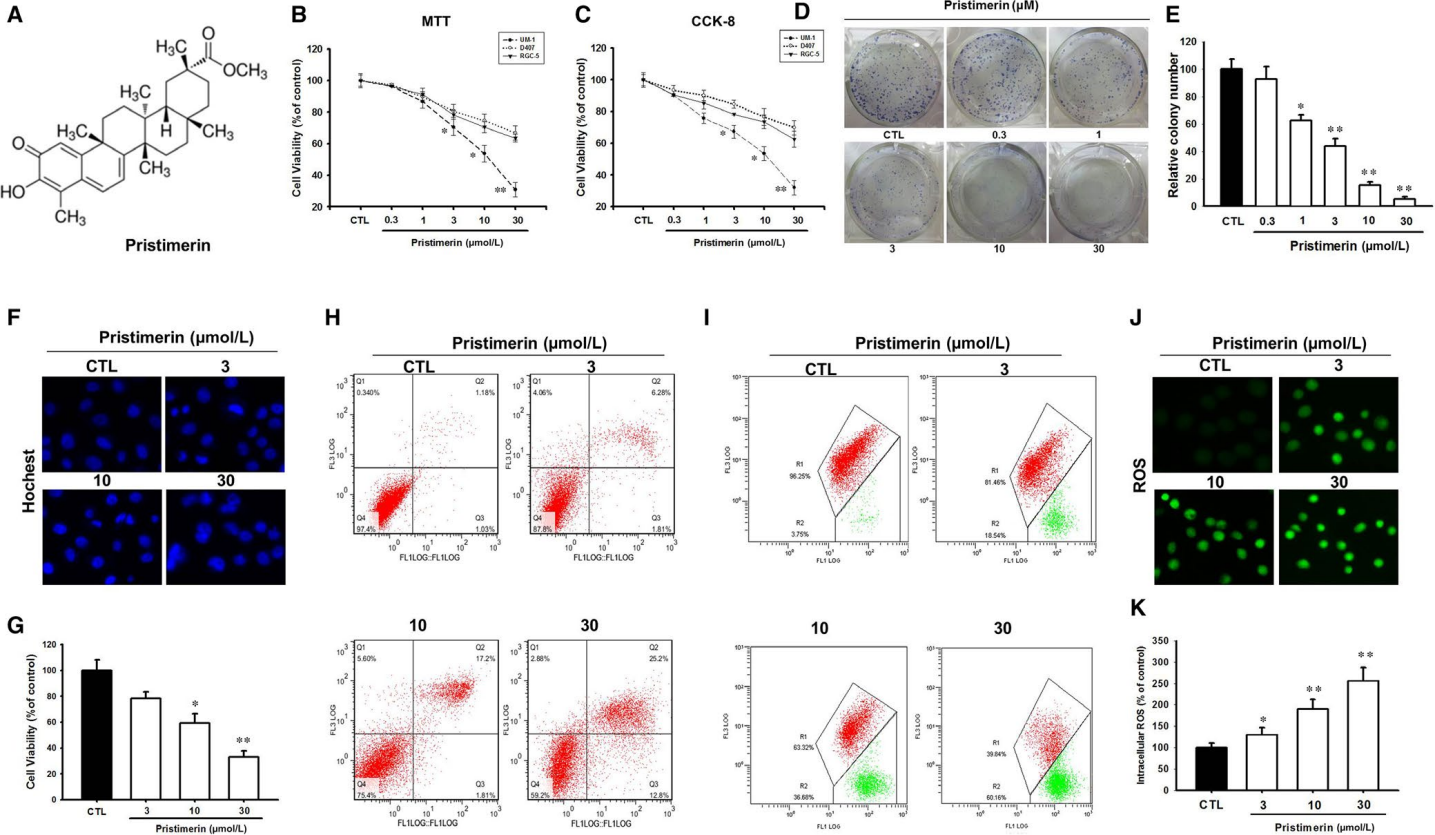
Pristimerin induced cytotoxicity in UM‐1 compared to RGC‐5 and D‐407 cells. (A) The chemical structure of Pristimerin; (B, C) UM‐1, RGC‐5 and D‐407 cells were treated for 24 h with different concentrations. Cell viability was determined by MTT (B) or CCK‐8 (C) assays; (D, E) UM‐1 cells were exposed to various concentrations for 14 d, and clonogenic assay was employed to detect cell reproductive death. UM‐1 cells were treated at indicated concentrations for 24 h, and then, the cells were stained with Hochest 33342 (F, G—apoptosis), FITC/PI (H, apoptosis), JC‐1 (I, mitochondrial membrane potential) or DCFH‐DA (J, K—ROS) followed by high‐content screening or flow cytometry. The data were analysed by Flowjo 7.6. The results represent mean ± SD of three separate experiments (^*^ *P* < .05, ^**^ *P* < .01 versus control group)

Pristimerin (Figure 1A) is a natural triterpenoid quinine compound, which is a traditional Chinese medicine isolated from the *Celastraceae* and *Hippocrateacea* plants. It has long been used as an anti‐malarial, anti‐inflammatory, anti‐oxidant and insecticide.^2^, ^3^ Recent studies have shown that Pristimerin potently induced anti‐proliferative and apoptosis activities in several human cancer cell lines, which originated from lung, breast, prostate, glioma, cervical, leukaemia and multiple myeloma tumours.^2^, ^4^, ^5^, ^6^, ^7^, ^8^ Induction of apoptotic cell death by Pristimerin involved with different mechanisms, including caspase activation, proteasomes inhibition, mitochondrial dysfunction and different molecular mechanisms involved in the suppression of anti‐apoptotic NF‐κB, Akt and MAP kinases.^9^, ^10^, ^11^ In addition, Pristimerin has been reported to activate the stress kinase, c‐Jun N‐terminal kinase(JNK) and the DNA damage sensor, poly (ADP‐ribose) polymerase‐1 (PARP‐1) through the generation of reactive oxygen species (ROS).^12^ Moreover, other studies indicated that Pristimerin inhibited cell cycle progression, tumour cell migration and angiogenesis.^5^, ^13^, ^14^, ^15^ Unfortunately, the cytotoxic effects and the molecular mechanism by which Pristimerin affects UM‐1 were poorly investigated and only one study reported that Pristimerin inhibited the malignant phenotypes of UM cells through inactivation of NF‐κB pathway.^16^ Here, we focus on the effect of Pristimerin on the PI3K/Akt signalling pathway in UM‐1 cells.

The PI3K/Akt pathway is a highly conserved central regulator of growth, proliferation, motility and metabolism. The genes and proteins of this pathway have been extensively studied and found to be widely activated in human cancers. Inhibition of this pathway has been shown to cause better regression of human tumours and has been evaluated in preclinical research and clinical trials.^17^, ^18^ Moreover, the activation PI3K/Akt pathway represents one of the most frequent genetic alterations found in human disease.^19^ PI3K/Akt pathway dysregulation has also been associated with resistance to conventional chemotherapy.^20^, ^21^

The FoxO transcription factors are the direct downstream target of the PI3K/AKT pathway, comprising four highly related members—FoxO1, FoxO3a, FoxO4 and FoxO6.^22^ The FoxO transcription factors are key regulators of diverse cell activities including cellular proliferation, differentiation, DNA repair, defence against oxidative stress, apoptosis and autophagy.^23^, ^24^, ^25^ They are also associated with multiple diseases, including cancer.^26^, ^27^ The activity of FoxO can be regulated post‐translational by various signalling pathways in which the Akt serves as an important regulator.^28^ Akt can directly phosphorylate FoxO transcription factors, promote their translocation into the cytoplasm from the nucleus and cause its functional inhibition.^29^ In the absence of active Akt, FoxO proteins are localized in the nucleus, where they regulate the transcription of genes related to cell cycle arrest, reactive oxygen species (ROS) detoxification and regulate expression levels of apoptotic proteins such as Bim, p27^Kip1^, p21 and PUMA.^30^, ^31^, ^32^

In the present study, we investigated Pristimerin‐induced pro‐apoptotic effects in the UM‐1 cells cultures in relation to PI3K/Akt/FOXO3a signalling pathway and found that Pristimerin induced apoptosis by inhibiting the PI3K/Akt/FoxO3a pathway. These results suggest that Pristimerin may be considered as a lead cytotoxic compound in the chemotherapy of uveal melanoma.

## 2 MATERIALS AND METHODS

### 2.1 Materials

Pristimerin, Annexin V‐FITC/PI, JC‐1 were purchased from Sigma‐Aldrich. Akt‐siRNA was obtained from Ribobio. Plasmid for FoxO3a, FoxO3a siRNA and Empty plasmid vectors N1 were kindly provided by Marten P. Smidt (Rudolf Magnus Institute of Neuroscience).

### 2.2 Cell cultures and transfection

The human uveal melanoma cell line UM‐1, the neuronal precursor cell line RGC‐5 and the D407 cells were obtained from Shanghai Bioleaf Biotech Co., Ltd. UM‐1 cell line clone was originally isolated from the uveal melanin tumour tissue. RGC‐5 cell line that displays mouse retinal ganglion progenitor cell characteristics correspond to mouse photoreceptor cell line 661W, providing a valuable tool to study pathogenesis of retinal neurodegenerative diseases. The cell lines were maintained in DMEM supplemented with 10% foetal bovine serum (FBS). UM‐1 cells were transfected with FoxO3a, FoxO3a siRNA or N1 (empty plasmid) plasmid using Lipofectamine 2000 according to the manufacturer's protocol.^25^ At 36 hours after transfection, cells were treated as indicated.

### 2.3 Cell viability assay

UM‐1 cells were plated at a density of 1 × 10^4^ per well in 96‐well plates, and the cells were treated with various concentrations of Pristimerin for 24h. Then, cell viability was assessed using MTT and CCK‐8 assays. All experiments were performed in 5 replicates and repeated for 3 times.

### 2.4 Clonogenic assay

UM‐1 cells were plated at a density of 200 cells/well in the 6‐well plates and then treated with various concentrations of Pristimerin. After 14 days, colonies were fixed with 4% paraformaldehyde, washed twice and stained with crystal violet (0.01% w/v). The colonies containing more than 50 cells were counted.

### 2.5 Hoechst 33342 staining for apoptosis

After treatment with Pristimerin, UM‐1 cells were fixed with 4% paraformaldehyde for 10 minutes. Then, the cells were washed twice with PBS and exposed to Hoechst 33342 (10 µg/mL) in PBS for10 minutes at room temperature. Chromatin staining pattern was analysed for individual cells by high content screening system (Array Scan VTI, Thermo Fisher Scientific, USA).

### 2.6 Annexin V‐FITC/PI assay

Apoptotic cells were detected using Annexin V‐FITC/PI double‐staining. Briefly, after treatment with Pristimerin with/without Akt siRNA or LY294002 (30 μM) for 24 hours, cells were harvested and stained with Annexin V‐FITC/PI according to the manufacturer's protocol. The apoptotic cells were analysed by fluorescence‐activated cell sorter (FACS), and the data were evaluated using the Flowjo 7.6.1 software.

### 2.7 Mitochondrial membrane potential assay

Mitochondrial membrane potential (∆ψ) was measured by the flow cytometer with JC‐1dye. The cells were analysed by flow cytometry, and the data were evaluated using the Flowjo 7.6.1 software.

### 2.8 Measurement of reactive oxygen species (ROS)

The production of ROS was evaluated using the fluorescent dye, 2,7‐dichlorodihydrofluorescein diacetate (DCFH‐DA, Sigma–Aldrich). For this assay, UM‐1 cells were treated with Pristimerin and then incubated with 50 µM DCFH‐DA for 30 minutes at 37°C. Subsequently, the fluorescence was then detected using a high content screening system (ArrayScanVTI, Thermo Fisher Scientific) using an excitation wavelength of 480 nm. The experiments were performed at least three times.

### 2.9 Cell cycle assay

We determined phases of cell cycle using flow cytometry. In brief, UM‐1 cells were seeded on 6‐well plates and treated with various concentrations of Pristimerin with/without LY294002 (30 µM) or Akt siRNA for 24 hours, and then, cells were collected after gentle trypsin treatment and fixed overnight in ice‐cold 70% ethanol, after which cells were treated with RNase A and stained with propidium iodide (PI). Finally, the cell cycle distribution was analysed by a flow cytometer and the date were calculated using the Flowjo 7.6.1 software. All experiments were performed in triplicate.

### 2.10 Transwell assay

For cell migration/ invasion assay, UM‐1 cells (1‐5 × 10^5^) were plated in serum‐free DMEM in the upper chamber on 8 μm pores filters coated with collagen (for cell migration) or Matrigel (for cell invasion) (BD Biosciences) and 500 μL DMEM with 10% FBS as chemoattractant was added to the bottom chamber. After 24 hours incubation, the migrated/invasive cells in the membrane were fixed in 4% formaldehyde and stained using 0.1% crystal violet for 30 minutes at room temperature. Finally, the number of migrated and invasive cells was counted in 8 randomly selected fields under inverted fluorescence microscope (×100 magnification). The mean number of cells per field was calculated as cell counts. All experiments were performed in triplicate.

### 2.11 Real‐time PCR

According to the manufacturer's instructions, total RNA was extracted with Trizol reagent (In Vitrogen). Reverse transcription was performed using Roche First Stand cDNA Synthesis Kit. PCRs were performed with the LightCycler^®^ 480 SYBR Green I using the following primers: FoxO3a (forward: 5′‐ CTCCCTACGCCAGTCTCCCAT ‐3′; reverse: 5′‐ TGAGTCCGAAGTGAGCAGGTCC ‐3′), Bim (forward: 5′‐ATAAGCTAAAGAGGCTGAAAGAG‐3′; reverse: 5′‐GAATGAAATGAGTCCCCAAAAC‐3′), p27^Kip1 ^(forward: 5′ –AAAAGCAACAGAAACCTATCCTCAC‐3′; reverse: 5′‐ ATTCAAAACTCCCAAGCACCTC‐3′), cyclin D1 (forward: 5′‐ CCCTCGGTGTCCTACTTCAAATGT‐3′; reverse: 5′‐ GGAAGCGGTCCAGGTAGTTCAT‐ 3′) and PRL‐19 (forward: 5′‐GAGACAAAGTGGGAGCCAGCGA‐3′; reverse: 5′‐ACCCTCCAGGAAGCGAGAATGC‐3′).

Quantitative analysis of cDNA amplification was assessed by incorporation of SYBR Green into the double‐stranded DNA. The expression of relative genes was examined by using the LightCycler 480 Instrument (Roche) and analysed with Relative Quantification LightCycler 480 SW 1.5.1 analysis software.

### 2.12 Western blotting

Western blotting was performed according to protocols routinely used in our laboratory, as described by Zheng et al.^33^ The protein bands were detected by ECL Blotting Detection Reagents. Blots were quantified using ImageJ analysis software. All experiments were performed in triplicate.

### 2.13 Reporter gene assay

UM‐1 cells were co‐transfected with FoxO3a‐pGL3 plasmid for firefly luciferase or the pGL2‐basic empty vector and pRL‐TK‐luc plasmid encoding for Renilla luciferase. Cells were incubated for 36h and treated with Pristimerin for additional 6, 12 or 24 hours, after which the activity of firefly, and Renilla luciferase was measured using the Dual Luciferase reporter assay system (Promega). Values for firefly luciferase activity were normalized to Renilla luciferase activity in the corresponding well. The experiments were repeated three times.

### 2.14 Small interfering RNAs

Akt siRNA (forward: 5′‐CCAUGAACGAGUUUGAGUA‐3′, reverse: 3′‐GGUACUUGCUCAAACUCAU‐5′) and negative control siRNA (forward: 5′‐UUCUCCGAACGUGUCACGUTT‐3′, reverse: 3′‐ACGUGACACGUUCGGAGAATT‐5′) were chemically synthesized by Ribio. Cells cultured in six‐well plates were transfected with 100 pM siRNA by Lipofectamine^®^ 3000 according to the manufacturer's instructions. Cells were then subjected to further treatment as requested by the protocol.

### 2.15 Immunofluorescence

After various treatments, UM‐1 cells were washed three times with PBS, fixed with 4% paraformaldehyde at room temperature for 30 minutes, permeabilized by 0.2% Triton X‐100 solution for 10 minutes, and then cells were blocked with 2% BSA for 30 minutes at room temperature. Subsequently, cells were incubated with primary polyclonal anti‐FOXO3a antibodies overnight at 4°C. Thereafter, cells were incubated with FITC ‐labelled secondary antibodies for 1 hour at room temperature, followed by staining with Hoechst 33342 to visualize the nucleus. The subcellular localization of FoxO3a was visualized under an inverted fluorescence microscope (Olympus).

### 2.16 Statistical analysis

The data are presented as the mean ± SEM of 3‐5 cultures, and experiments were performed at least three times. Statistical comparison between groups was performed by ANOVA, followed two‐sided *t*‐test using SPSS 13.0 program. Differences were considered significant when *P* < .05.

## 3 RESULTS

### 3.1 Pristimerin induced UM‐1 cells apoptotic death

To determine the sensitivity of the cytotoxic effect of Pristimerin in UM‐1 cells, by comparison to a retinal ganglion derived neuronal cell line (RGC‐5) and a human retinal pigment epithelial cell line (D407), the cells were treated with Pristimerin for 24h and the viability was estimated using MTT and CCK‐8 assays (Figure 1B,C). We found that Pristimerin inhibited UM‐1 cell viability in a dose‐dependent manner, reaching 80% cell death at 30 µM. However, the neuronal and epithelial cell cultures RGC‐5 and D407 were more resistant than UM‐1 cells towards the cytotoxic effect of Pristimerin, indicating only marginal, 20% cytotoxicity, upon treatment with 30 µM Pristimerin.

To investigate the effect of Pristimerin on colony formation, UM‐1 cells were incubated with various doses of Pristimerin for 10‐14 days and colonies containing more than 50 cells were counted. The results showed the Pristimerin inhibited colony formation by UM‐1 cells in a concentration‐dependent manner, with an effective concentration 50% (EC_50_) of 2 µM (Figure 1D,E). To observe the morphological characteristics of Pristimerin‐induced cell death, UM‐1 cells were exposed to different concentrations of Pristimerin for 24 hours and the apoptotic cells were stained with Hoechst 33342 followed by high content screening system analysis. Pristimerin in a dose‐dependent manner induced cell apoptosis, indicated by reduction of cell volume, bright staining and condensed or fragmented nuclei (Figure 1F,G).

In another approach, to confirm that Pristimerin induced apoptosis, UM‐1 cells were stained with FITC/PI and apoptosis was evaluated by FACS. The results showed that Pristimerin induced cell apoptosis in a concentration‐dependent manner (Figure 1H), these findings consistent with Hoechst 33342 staining experiments.

The decline of mitochondrial transmembrane potential is another important indicator in early apoptosis. To assess a potential effect of Pristimerin on mitochondrial transmembrane potential, UM‐1 cells were incubated with JC‐1 and the change of fluorescence from red to green, reflecting the decline of the membrane potential, was measured. As shown in Figure 1I, Pristimerin induced a decline of mitochondrial membrane potential in a dose‐dependent fashion. Moreover, to assess whether the mitochondrial dysfunction was correlated to increased ROS level, the intracellular ROS level was evaluated using high content screening system. UM‐1 cells were treated with different concentrations of Pristimerin, stained with DCFH‐DA and the percentage of stained cells was measured compared with control. The results indicated that intracellular ROS level was increased in a concentration‐dependent manner in UM‐1 cells (Figure 1J,K), providing an explanation to the findings of Pristimerin‐induced mitochondrial dysfunction. Cumulatively, the findings described in Figure 1 indicated that the UM‐1 cells are more sensitive than neuronal and epithelial cells towards the cytotoxic‐apoptotic cell death effect of Pristimerin.

### 3.2 Inhibitory effect of Pristimerin on cell cycle of UM‐1

**FIGURE 2:**
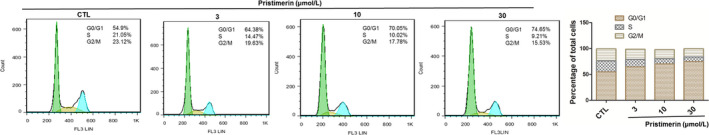
Pristimerin induced cell cycle arrest of UM‐1 cells. UM‐1 cells were treated at indicated concentrations for 24 h; cells were collected and stained with propidium iodide (PI), and then analysed using the flow cytometer. Cell cycle distribution (%) in each cell cycle phase was calculated using Flowjo 7.6. The results represent mean ± SD of three separate experiments

To estimate the effects of Pristimerin on the cell cycle of UM‐1 cells, the cultures UM‐1 cells were treated with various concentrations of Pristimerin for 24 hours, and the cell cycle distribution was determined by flow cytometry. As shown in Figure 2, Pristimerin induced in a dose‐dependent manner a significant accumulation of a cell population in G0/G1phase, in parallel to a reduction of the distribution of other cell population in S‐G2/M phase of the cell cycle. These findings suggested that Pristimerin inhibited the G0/G1 to S cell cycle phase transition, in direct correlation with the significant apoptotic cell death of UM‐1 cells (Figure 1). These findings highlight a rational for further development of Pristimerin to treat human uveal melanoma tumours.

### 3.3 Pristimerin inhibited migration and invasion of UM‐1 cells

**FIGURE 3:**
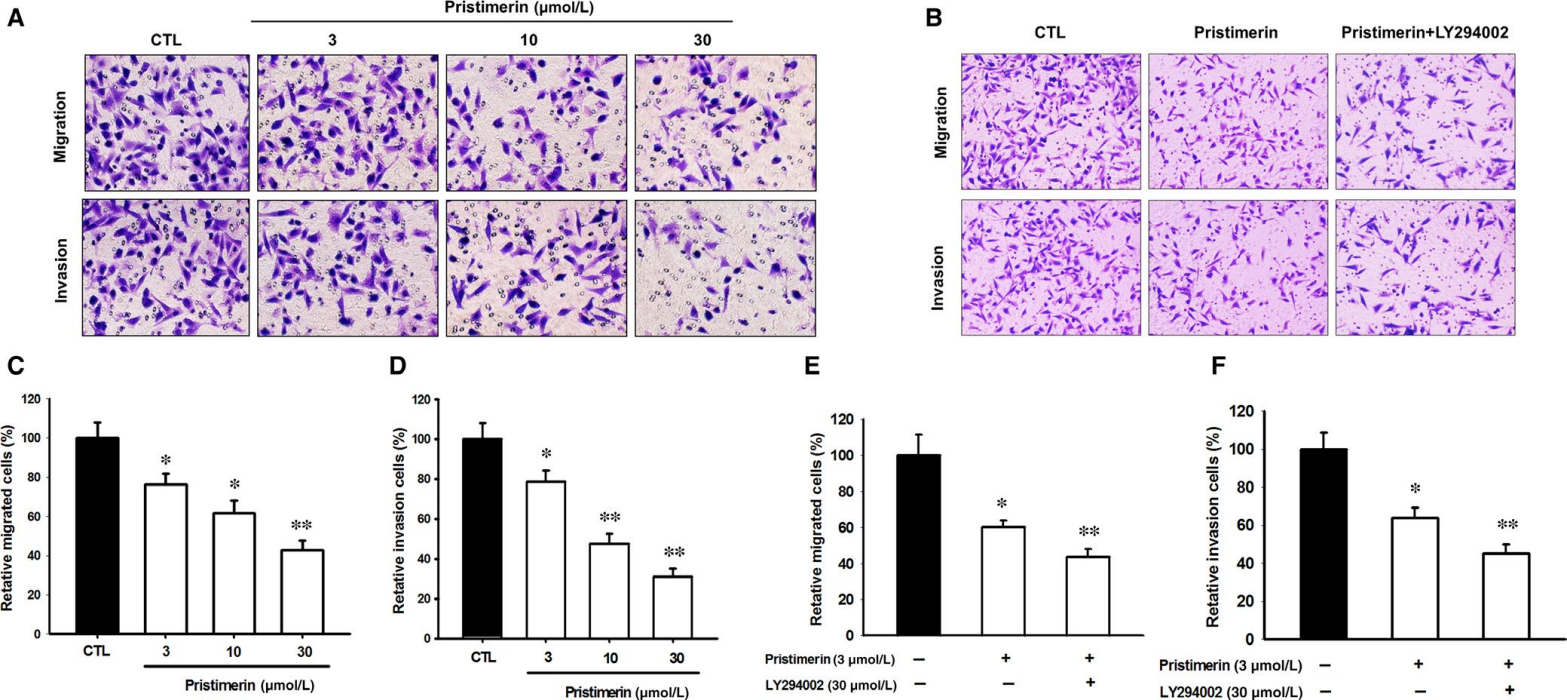
Pristimerin inhibited migration of UM‐1 cells. UM‐1 cells were treated at different concentrations (A) or with 3 µM for 24 h with or without pretreatment with LY294002 (30 μM, 1 h) (B), and the transwell assay was employed to measure migration on collagen and invasion on Matrigel‐coated filters. Upper A‐B figures represent stained cells on the lower surface of the filters. Graphs represent quantification (%) of uveal melanoma cells that migrate (C, E) and invade (D, F) through transwell filter. The results represent mean ± SD of three independent experiments (^*^ *P* < .05 versus control group; ^**^ *P* < .01 versus control group)

UM‐1 cells were treated with different concentrations of Pristimerin, and the transwell assay was used to determine their migration and invasion properties. In the cell migration assay (Figure 3A,C), we found that Pristimerin, in a dose‐dependent way, significantly inhibited that cells' ability to migrate through a collagen matrix with an estimate EC_50_ of 25 µM. In Matrigel invasion assay, Pristimerin treatment also markedly reduced the percentage of invading cells with an estimated EC_50_ of 10 µM (Figure 3A,D). These results suggested that Pristimerin might be effective in the treatment of uveal melanoma through inhibition of tumour cells migration and invasion.

### 3.4 The effect of Pristimerin on the Akt/FoxO3a pathway and cleaved caspase‐3, PARP, Bax and Bcl‐2 expression in UM‐1 cells

**FIGURE 4:**
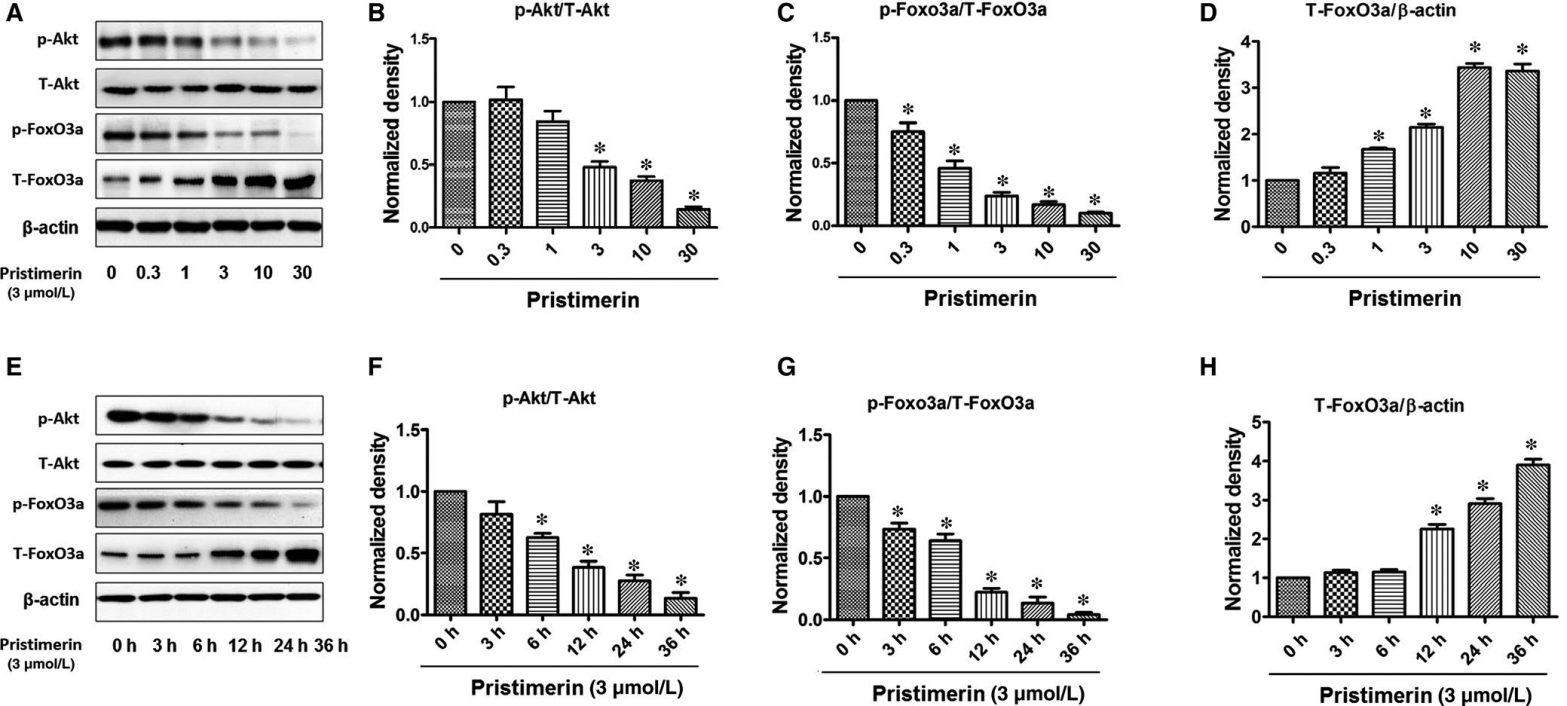
Pristimerin induced dose‐response and time course inhibition of PI3K/Akt/FoxO3a phosphorylation in UM‐1 cells. (A, E) UM‐1 cells were treated at different concentrations or different times, and Western blotting was performed to measure phosphorylations compared to that in control untreated cultures. (B‐D) and (F‐H) Quantification of phospho‐immunoreactivity was performed by densitometry. The results represent mean ± SD of three independent experiments (^*^ *P* < .05 versus control group; ^**^ *P* < .01 versus control group)

**FIGURE 5:**
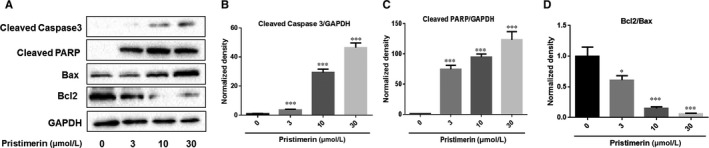
Effects of Pristimerin on the expression of cleaved caspase‐3, PARP, Bax and Bcl‐2. A‐Um‐1 cells were treated with Pristimerin at indicated concentrations for 24 h, and the protein expression levels of caspase‐3, PARP, Bax and Bcl‐2 were measured using Western blotting. (B‐D)‐ Densitometric analysis of the immunoblots expressed as percentage of control. Results are shown as the mean ± SD, ^***^ *P* < .01 vs control group

To determine whether Pristimerin could influence Akt/FoxO3a signalling pathway in UM‐1 cells, UM‐1 cells were treated with Pristimerin at different concentrations (Figure 4A‐D) or different periods of time (Figure 4E‐H) and Western blotting was performed to measure phosphorylations of Akt and FoxO3a. We have noted a significant reduction of phosphorylated‐AKT at Ser473 upon treatment with concentrations of Pristimerin higher than 3 µM and after 6 hours of treatment, while the level of its total protein remained stable throughout all the time points of treatment with the different Pristimerin concentrations (Figure 4A,B,E,F). Consequently, the phosphorylation level of transcriptional factor FoxO3a at Ser253 was also found to be significantly inhibited at 3‐36 h from the start of treatment with different Pristimerin concentrations (Figure 4A,C,E,G). Interestingly, the level of total FoxO3a significantly increased from 12‐36 h upon treatment with Pristimerin concentrations from 1 to 30 µM (Figure 4A,E,D,H). Meanwhile, we also found that Pristimerin in a dose‐dependent fashion increased cleaved caspase‐3, PARP and Bax levels of expression, but reduced the level of expression of Bcl‐2 and as a consequence the ratio of Bcl‐2/Bax was also decreased (Figure 5). These results indicate an apparent direct correlation among Pristimerin‐induced cytotoxic effects and inhibition of Akt/FoxO3a phosphorylation activities and cleaved caspase‐3, PARP, Bax and Bcl‐2 expression.

### 3.5 PI3K/Akt inhibitors and Akt siRNA additively potentiated the cytotoxic effects of Pristimerin in UM‐1 cells

**FIGURE 6:**
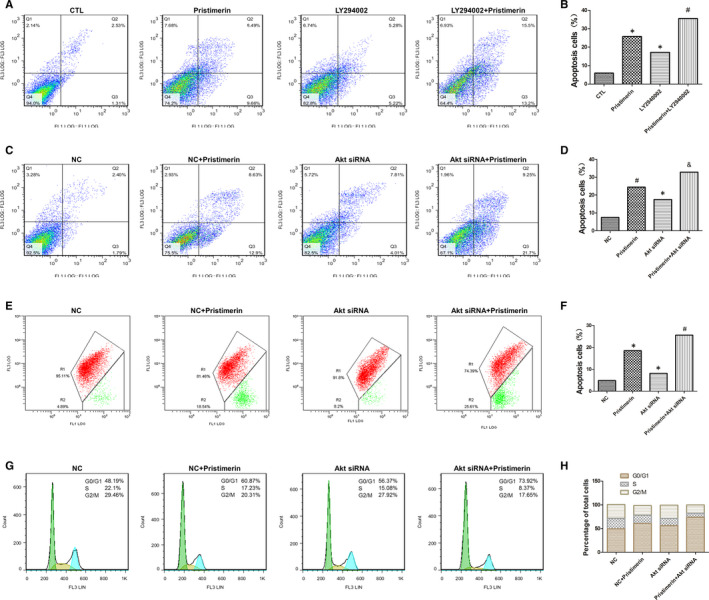
Effects of PI3K/Akt inhibition on Pristimerin‐induced cell cycle distribution, cells apoptosis and mitochondrial membrane potential in UM‐1 cell. (A‐D) apoptosis; (E, F) mitochondrial membrane potential; (G, H) cell cycle distribution. UM‐1 cells were first pretreated with 30 µM LY294002 for 1 h or 10 µM Akt siRNA for 36 h and thereafter exposed for 24 h to 3 µM Pristimerin. Thereafter, the cultures UM‐1 cells were analysed by flow cytometry upon staining with FITC/PI (A, C) or JC‐1 (E). The results represent mean ± SD of three independent experiments (^*^ *P* < .05 versus control group; ^#^ *P* < .05 versus Pristimerin group)

To explore the contribution of PI3K/Akt inhibition to the cytotoxic effects of Pristimerin, UM‐1 cells were either pretreated with LY294002, a potent, reversible, morpholine‐containing inhibitor of phosphoinositide 3‐kinases (PI3K) which phosphorylates and activates Akt, or transfected with Akt siRNA, before exposure to Pristimerin. The apoptotic cell death (Figure 6A‐D), the mitochondrial membrane potential (Figure 6E,F) and cell cycle distribution (Figure 6G,H) of the UM‐1 cells following the different treatments was measured by FACS. When used in combination with Pristimerin, the PI3K/Akt inhibitors potentiated by 30% the percentage of apoptotic cells, with similar efficacy of LY294002 and Akt siRNA (Figure 6A‐D). This conclusion was further confirmed by measuring the Pristimerin‐induced reduction of the mitochondrial membrane potential (Figure 6E,F) and the percentage of cells accumulating in the G0/G1phase of the cell cycle (Figure 6G,H). Moreover, Pristimerin induced inhibition of UM‐1 cells’ migration and invasion also indicates that PI3K/Akt inhibition contributed about 20% additive potentiation to Pristimerin‐induced effects (Figure 3B,E,F), as previously reported with MET tyrosine kinase receptor inhibitor.^34^

### 3.6 PI3K/Akt/FoxO3a inhibitors differentially modulated protein expression of cell cycle and apoptosis‐associated proteins in UM‐1 cells induced by Pristimerin

**FIGURE 7:**
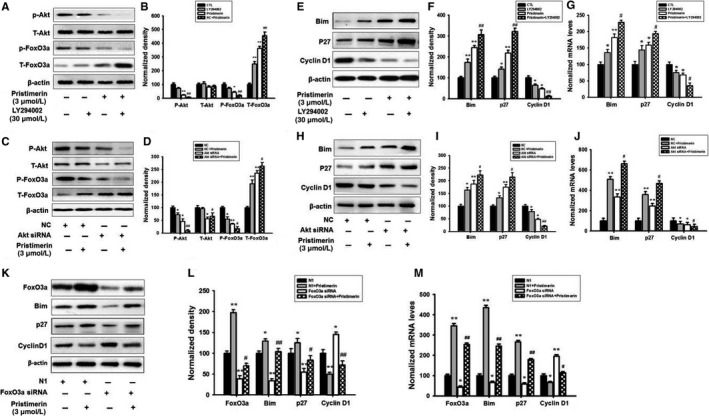
Apparent correlations between Pristimerin‐induced cell cycle and apoptosis‐associated proteins or inhibition of PI3K/Akt/FoxO3a phosphorylation, in UM‐1 cells. Um‐1 cells pretreated with LY294002 (A, B) or Akt siRNA (C, D) or FoxO3a siRNA (K, L) were treated with Pristimerin for 24 h and the protein and gene expression of Akt, FoxO3a, Bim, p27Kip1 and Cyclin D1 were measured using Western blotting and qPCR (G, J, M). Quantification of immunoreactivity was performed using densitometry analysis. The results represent mean ± SD of three independent experiments (^*^ *P* < .05 or ^**^ *P* < .01 versus control group; ^#^ *P* < .05 or ^##^ *P* < .01 versus Pristimerin group)

In the next step, we investigated the effects of PI3K/Akt and FoxO3a inhibitors on Pristimerin effects on phosphorylation and expression protein level of FoxO3a, a transcriptional factor that regulates gene expression, essential for cell cycle arrest and apoptosis in its dephosphorylated form in a nuclear location.^35^, ^36^ Figure 7A‐D indicated that both LY294002 and Akt siRNA treatments strongly potentiated the inhibitory effect of 3 μM Pristimerin on Akt (Ser473) and FoxO3a (Ser253) phosphorylations, when compared to the respective control groups. These effects were correlated with a significant, Pristimerin induced decrease of FoxO3a (Ser253) phosphorylation but increase of total FoxO3a protein expression level. Likewise, inhibition of FoxO3a phosphorylation was followed by up‐regulation of mRNA and protein level of the pro‐apoptotic molecules BIM and p27^Kip1^ and down‐regulation of anti‐apoptotic protein cyclin D1, effects potentiated by LY294002 and Akt siRNA pretreatment (Figure 7E‐J). When cultures were treated with Pristimerin, the mRNA levels of FOXO3a significantly changed (Figure 7M). Complementary to these results, FoxO3a knockdown experiments, using a selective siRNA approach, indicated that Pristimerin effect was partially abolished in FoxO3a knockdown UM‐1 cell, which indicated that knockdown of FOXO3a partially attenuated the Pristimerin‐induced apoptosis (Figure 7 K‐M). Cumulatively, these experiments indicate that Primisterin induced differential expression of various PI3K/Akt/FoxO3a downstream cell cycle and apoptosis associated protein substrates, an effect which was concentration and time dependent correlated to Pristimerin‐induced apoptotic cell death of UM‐1 cell cultures.

### 3.7 Pristimerin inhibited cytoplasmic‐nuclear shuttling and phosphorylation activity of FoxO3a

**FIGURE 8:**
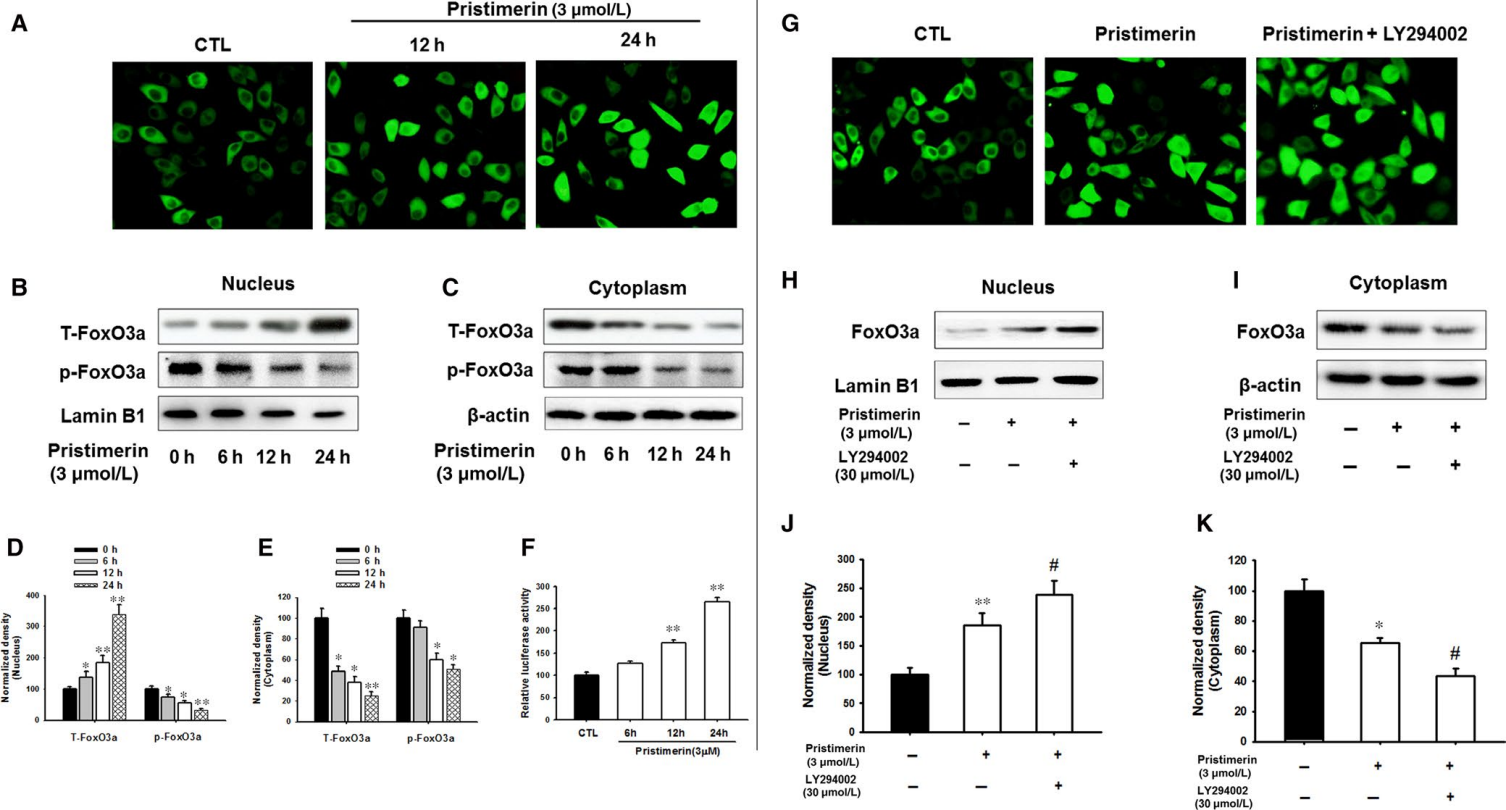
Pristimerin inhibited FoxO3a nuclear‐cytoplasm shuttling and phosphorylation activity in UM‐1 cells. (A, G) Following FoxO3a stable transfection, UM‐1 cells were treated with Pristimerin for the indicated time periods with or without 30µM LY294002 and the nuclear translocation of anti‐FoxO3a was measured by immunofluorescence. (B, C, H, I) UM‐1 cells were treated with Pristimerin for the indicated time periods with or without 30 µM LY294002, and the level of FoxO3a in the nucleus and cytoplasm was measure by Western blotting. Lamin B1 and β‐actin were used as markers of the nucleus and cytoplasm, respectively. (D, E, J, K) Quantification of immunoreactivity performed by densitometric analysis. (F) The cultures cells were transfected with FoxO3a‐pGL3 luciferase plasmid or pRL‐TK‐luc plasmid, followed by treatment with or without 3 µM Pristimerin for the indicated periods of time, after which, the transactivation of FoxO3a was measured using Dual‐luciferase reporter assay. Data represent mean ± SD of three independent experiments, (^*^ *P* < .05, ^**^ *P* < .01 versus control group; ^#^ *P* < .05 versus Pristimerin group)

We used immunofluorescence microscopy to observe the shuttling of FoxO3a in the control compared with the Primistirin‐treated in UM‐1 cells (Figure 8A,G). Akt phosphorylates FoxO3a and promoted its retention in the cytoplasm, thus prevented cell regulatory actions which took place in the nucleus. In contrast, Akt inhibition induced FoxO3a dephosphorylation and promoted its translocation from the cytoplasm into the nucleus.^35^, ^37^ To explore the effect of Pristimerin on cellular shuttling of FoxO3a, UM‐1 cells were transfected with GFP‐FoxO3a. After 6h, UM‐1 cells were treated with 3 µM Pristimerin for 12 and 24 hours, respectively (Figure 8A), with or without treatment with PI3K inhibitor LY294002 (30 µM) (Figure 8G). Thereafter, the subcellular localization of FoxO3a was determined by immunofluorescence microscopy. The expression of FoxO3a in nuclear and cytoplasmic extracts of different treatment groups was also assessed by Western blotting (Figure 8B‐E,H‐K). The immunofluorescence micrographs unambiguously indicated that Pristimerin significantly and progressively enhanced, with a time course of 12‐24 hours and by 3.4‐fold, nuclear level of FoxO3a protein in UM‐1 cells, an effect potentiated by LY294002 (Figure 8A,G). To further corroborate these findings, we extracted cytoplasmic and nuclear proteins and the level of non‐phosphorylated and phosphorylated FoxO3a was measured in both control and Pristimerin‐treated group by Western blotting using respective antibodies and considering Lamin B1 and β‐actin as markers of nucleus and cytoplasm, respectively. We found that Pristimerin increased the nuclear levels of FoxO3a protein but decreased its levels of phosphorylation in a time‐dependent manner (Figure 8B,D), an effect potentiated by LY294002 (Figure 8H‐K). Furthermore, the reporter gene assay indicated that Pristimerin promoted FoxO3a transactivation activity (Figure 8F). Taken together, these results indicate an apparent correlation between Pristimerin‐induced apoptotic cell death and inhibition of PI3K/Akt phosphorylation and FoxO3a nuclear‐cytoplasm shuttling inhibition and transactivation.

## 4 DISCUSSION

Uveal melanoma (UM) is considered a rare disease, but in fact, it is the most common primary intraocular malignant tumour in adults.^38^ Paradoxically, even combined with surgery, chemotherapy and radiotherapy, the median survival time of patients is did not improve significantly.^39^ Natural products derived from medicinal plants have been used since ancient times for the treatment of many diseases and have an important contribution to the discovery and development of new drugs with therapeutic potential against tumours.^40^, ^41^ Pristimerin, a triterpenoid quinone methide molecule, is characterized by beneficial pharmacological properties such as anti‐inflammatory, anti‐oxidant, anti‐tumour, anti‐malaria and anti‐microbial activities. However, Pristimerin‐induced cell death in UM‐1 cells was poorly investigated. In the present study, we found that Pristimerin induced a pro‐apoptotic effect in the UM‐1 cells through modulation of the PI3K/Akt/FoxO3a signalling pathway. We found that Pristimerin increased ROS, decreased the mitochondrial membrane potential, promoted accumulation of cells in G0/G1 phase of the cell cycle and induced apoptotic cell death.

In recent years, it has reported that Pristimerin could affect many tumour‐related processes, such as autophagy, apoptosis, vasculogenesis, migration and invasion, and drug resistance.^42^ In human breast cancer cells, Pristimerin‐triggered apoptosis through caspase activation, which could be completely prevented by benzyloxycarbonyl Val‐Ala‐Asp‐fluoromethyl ketone, a pan‐caspase inhibitor.^10^ In pancreatic cancer, Pristimerin induced cell apoptosis by inhibition of NF‐kB.^43^ In prostate cancer cells, Pristimerin induced cell death by effective proteasome inhibition.^5^ However, the molecular mechanisms involved in the cytotoxic effects of Pristimerin in tumour cells in general and uveal melanoma tumour cells in particular, have not been fully explored.

In the present study, we found that Pristimerin inhibited of UM‐1 cells proliferation, accumulation of cells in the G0/G1 phase of the cell cycle and decreased survival. Moreover, Pristimerin stimulated UM‐1 apoptotic cell death expressed by nuclear condensation and fragmentation and increased Annexin V staining, representing binding to phosphatidylserine, which is increased in the plasma membrane of apoptotic cells. Therefore, Pristimerin induction of UM‐1 cell‐cycle arrest and apoptosis resemble some of the anti‐tumour effects of conventional chemotherapeutic agents. We also found that UM‐1 cells are more sensitive towards the apoptotic cell death effects of Pristimerin than retinal RGC‐5 ganglion and retinal D407 pigment epithelial cell models commonly evaluated in cytotoxic studies of chemotherapy.^44^ These findings suggested that upon systemic or intravitreal delivery of cytotoxic doses of Pristimerin, less retinal toxicity is expected.

Mitochondria have been shown to play a key role in the apoptotic process due to the release of the pro‐apoptotic proteins.^45^ Pristimerin was found to induce mitochondria depolarization^46^ and in breast tumour cells caused a rapid release of cytochrome *c*, triggering caspase activation and decrease of the mitochondrial membrane potential.^10^ Here, we confirmed these findings since treatment of UM‐1 cells with Pristimerin indicated decrease of mitochondrial membrane potential in correlation to the cytotoxic effect. Mitochondrial reactive oxygen species (ROS) production play a crucial role in induction of both intrinsic and extrinsic apoptotic cell death.^47^ Many studies have showed that some anticancer agents, such as Epirubicin and Daunomycin, induced apoptosis in part with the generation of ROS and the disruption of redox homeostasis.^48^ The increase production of ROS may lead directly to the mitochondrial permeability transition activation and induce the loss of mitochondrial membrane potential.^49^ In line with this concept, Pristimerin induced mitochondrial cell death through ROS generation in cervical tumour cells.^7^, ^12^ However, the generation of ROS in breast cancer cells was not affected by Pristimerin,^10^, ^12^ indicating differences between tumour types. Here, we found that Pristimerin stimulated production of ROS in UM‐1 cells, in correlation with the decrease in the mitochondrial membrane potential, emphasizing the role of ROS‐mediated mitochondrial dysfunction in its cytotoxic effect. These data are reminiscent of previous reports indicating that Pristimerin anticancer effects involve ROS‐mediated mitochondrial dysfunction.^8^, ^12^, ^50^

The PI3K/Akt signalling pathway is necessary for cell growth and survival in many human cancers and is also activated in uveal melanoma.^51^, ^52^ Akt promotes cell survival by phosphorylating and inhibiting Forkhead transcription factor (FoxO), an important downstream substrate, known as important gene regulator and tumour suppressor.^53^, ^54^, ^55^ Our study indicated that Pristimerin inhibited Akt, and Akt inhibition resulted with FoxO3a dephosphorylation which led to FoxO3a accumulation in the nucleus of the UM‐1 cells. This is a key causal and temporal event responsible for Pristimerin‐induced inhibition of proliferation, cell‐cycle arrest and apoptosis in the UM‐1 cells. By virtue of inhibiting PI3K/Akt/FoxO3a pathway, Pristimerin could be a promising therapeutic option for UM.

Taken together, the following findings indicated that Pristimerin‐induced apoptosis in UM‐1 cells is in a direct correlation with inhibition of PI3K/Akt/FoxO3a pathway in UM‐1 cells: (a) The Pristimerin in a dose‐and time‐dependent manner inhibited PI3K/Akt signalling, changed the mRNA levels of FOXO3a and activated FoxO3a protein; (b) Inhibition of Akt strongly potentiated the inhibitory effect of Pristimerin on Akt (Ser473) and FoxO3a (Ser253) phosphorylation; (c) Pristimerin‐induced inhibition of UM‐1 cells’ migration and invasion was potentiated by PI3K/Akt inhibition; (d) Pristimerin induced up‐regulation of the pro‐apoptotic molecules BIM, p27^Kip1^, cleaved caspase‐3, PARP and Bax and down‐regulation of anti‐apoptotic protein cyclin D1 and Bcl‐2, effects potentiated by Akt inhibition; (e) Pristimerin‐induced FoxO3a translocation into the nucleus by inhibition of its cytoplasmic‐nuclear shuttling and phosphorylation activity.

In conclusion, we propose that Pristimerin induced apoptosis by inhibiting the PI3K/Akt/FoxO3a signalling pathway in UM‐1 cells. These in vitro findings propose considerations of Pristimerin as a lead cytotoxic compound in the chemotherapy of uveal melanoma. Further studies in animal models as well as clinical studies are required to characterize the therapeutic potential of Pristimerin towards UM tumours.

## CONFLICT OF INTEREST

The authors declare no conflict of interest.

## AUTHOR CONTRIBUTIONS

Fenxia Yan and Rifang Liao performed the experiments and drafted the manuscript. Marta Silva and Shuai Li performed part of experiments. Tangming Peng and Philip Lazarovici revised the manuscript. Wenhua Zheng conceived the hypothesis, designed the experiments and revised the manuscript. All authors read and approved the final manuscript.

## Data Availability

The data are available from the corresponding author on reasonable request.
